# Assessment of the Role of Metabolic Determinants on the Relationship between Insulin Sensitivity and Secretion

**DOI:** 10.1371/journal.pone.0168352

**Published:** 2016-12-21

**Authors:** Jose E. Galgani, Carmen Gómez, Maria L. Mizgier, Juan Gutierrez, Jose L. Santos, Pablo Olmos, Andrea Mari

**Affiliations:** 1 Departamento de Nutrición, Diabetes y Metabolismo, Escuela de Medicina, Pontificia Universidad Católica de Chile, Santiago, Chile; 2 UDA-Ciencias de la Salud, Carrera de Nutrición y Dietética, Escuela de Medicina, Pontificia Universidad Católica de Chile, Santiago, Chile; 3 Istituto di Neuroscienze, Consiglio Nazionale delle Ricerche, Padova, Italy; Louisiana State University, UNITED STATES

## Abstract

**Background:**

Insulin secretion correlates inversely with insulin sensitivity, which may suggest the existence of a crosstalk between peripheral organs and pancreas. Such interaction might be mediated through glucose oxidation that may drive the release of circulating factors with action on insulin secretion.

**Aim:**

To evaluate the association between whole-body carbohydrate oxidation and circulating factors with insulin secretion to consecutive oral glucose loading in non-diabetic individuals.

**Methods:**

Carbohydrate oxidation was measured after an overnight fast and for 6 hours after two 3-h apart 75-g oral glucose tolerance tests (OGTT) in 53 participants (24/29 males/females; 34±9 y; 27±4 kg/m^2^). Insulin secretion was estimated by deconvolution of serum C-peptide concentration, β cell function by mathematical modelling and insulin sensitivity from an OGTT. Circulating lactate, free-fatty acids (FFA) and candidate chemokines were assessed before and after OGTT. The effect of recombinant RANTES (regulated on activation, normal T cell expressed and secreted) and IL8 (interleukin 8) on insulin secretion from isolated mice islets was also measured.

**Results:**

Carbohydrate oxidation assessed over the 6-h period did not relate with insulin secretion (r = -0.11; p = 0.45) or β cell function indexes. Circulating lactate and FFA showed no association with 6-h insulin secretion. Circulating chemokines concentration increased upon oral glucose stimulation. Insulin secretion associated with plasma IL6 (r = 0.35; p<0.05), RANTES (r = 0.30; p<0.05) and IL8 (r = 0.41; p<0.05) determined at 60 min OGTT. IL8 was independently associated with *in vivo* insulin secretion; however, it did not affect *in vitro* insulin secretion.

**Conclusion:**

Whole-body carbohydrate oxidation appears to have no influence on insulin secretion or putative circulating mediators. IL8 may be a potential factor influencing insulin secretion.

## Introduction

Glucose homeostasis requires of a complex interplay in which pancreatic β cells sense glucose concentration in order to release an appropriate amount of insulin. In addition, the extent at which insulin is secreted takes into account the degree of systemic insulin sensitivity [1]. Considering that insulin sensitivity is mostly determined by peripheral tissues, one can hypothesize that an inter-organ humoral communication between these tissues and pancreas takes place. Due to the fact that skeletal muscle is the main site of glucose disposal in postprandial [2] and steady-state insulin-stimulated [3] conditions, and also the largest tissue in non-obese individuals [4], one can propose that skeletal muscle interacts with pancreas [5]. It is well known that skeletal muscle may secrete several soluble factors (e.g. myokines) [6,7]. Some of them may be sensitive to changes in skeletal muscle insulin sensitivity or glucose metabolism [5]. Then, one or a combination of these circulating factors might influence insulin secretion. Support for this hypothesis came from two skeletal muscle-specific genetic mice models, which are characterized by altered skeletal muscle glucose metabolism and abnormal *in vivo* insulin secretion [8,9]. Taken together, insulin secretion appears to be determined at central (β cells) and possibly peripheral (skeletal muscle, liver and adipose tissue) level.

In humans, glucose-stimulated insulin secretion assessed following a 4-h isoglycemic-hyperinsulinemic clamp was higher when compared with a 4-h saline infusion [10,11]. This finding was considered to be in line with an earlier *in vitro* study that found higher β cell insulin secretion after insulin treatment [12]. However, that finding has not been consistently reported among studies. Indeed, most of the studies show that insulin inhibits *in vitro* insulin secretion [13–16]. Therefore, enhanced glucose-stimulated insulin secretion observed after insulin pre-exposure may have an alternative explanation. Under the context of an inter-organ communication and considering that skeletal muscle glucose metabolism is greatly influenced in a glucose clamp, we propose that a skeletal muscle-derived circulating factor, which can be sensitive to changes in glucose metabolism, may play a role driving insulin secretion [5]. In line with this idea, we recently reported that 24-h carbohydrate oxidation (a main metabolic fate of glucose) related with 24-h insulin secretion (by urinary C-peptide excretion) even after controlling for insulin sensitivity [17].

Considering the fact that metabolic response to nutrients after an overnight fasting differs when compared with a second nutrient stimulation [18], in this study, we further explored the association of carbohydrate oxidation with insulin secretion rate after two consecutive oral glucose loadings. In addition, the association of carbohydrate oxidation with potential circulating mediators, including lactate, free-fatty acids [FFA] and molecules known to be secreted from skeletal muscle (interleukin 6 and 8 [IL6 and 8]; fractalkine; regulated on activation, normal T cell expressed and secreted [RANTES] and monocyte chemoattractant protein 1[MCP1]) was determined. Finally, the correlation between these factors and insulin secretion and β cell function was also evaluated.

## Methods

### Subjects

Healthy (by physical examination and routine laboratory tests), non-diabetic participants (24 males/29 females) were recruited by advertising (Table 1). They had stable body weight (change <2 kg over the past 3 months) and none performed regular physical activity (<60 min/week) or took medications except oral contraceptives in some females (12 out of 29). The protocol was approved by the Ethical Board at Pontificia Universidad Católica de Chile, and participants provided written informed consent. Body fat mass was measured by electrical bioimpedance (Inbody 230, Biospace. Seoul, Korea). Fat-free mass (FFM) was calculated as the difference between body mass and fat mass.

**Table 1:** Characteristics of participants.

	Mean ± SD	Range
Males/Females (n)	24/29	–
Age (years)	33.9 ± 8.7	21.0–54.0
Body mass (kg)	74.1 ± 14.1	50.6–114.9
Height (m)	1.67 ± 0.08	1.54–1.85
Body mass index (kg/m^2^)	26.5 ± 3.8	20.5–34.7
Fat mass (%)	31.0 ± 7.7	16.8–47.2
Glycated hemoglobin (%)	5.2 ± 0.3	4.2–5.7
Cholesterol (mg/dL)	175 ± 33	116–246
Triglycerides (mg/dL)	101 ± 49	25–267

### Animals

Male C57BL6/J mice were housed in a temperature- and light-controlled room and were allowed to consume standard chow (Prolab RMH3000, Labdiet) and water *ad libitum*. These studies were carried out in accordance with the National Research Council (NRC) publication Guide for Care and Use of Laboratory Animals (copyright 1996, National Academy of Science). The protocol was approved by the Ethics Committee for Animal Welfare from the School of Medicine of the Pontifical Catholic University of Chile.

### Experimental design

Participants were instructed to avoid vigorous physical activity for the day preceding metabolic testing and maintain their customary dietary pattern. In addition, alcohol, tobacco and caffeine-containing drinks were not allowed for the last 12 hours before testing. On the testing day, an intravascular cannula was inserted into an antecubital vein of overnight fasted individuals. Then, they rested for 30 min in supine position under thermoneutral and quiet conditions prior to gas exchange determination (VMax Encore 29n; SensorMedics Co. Yorba Linda, CA) for 20 min. In that period, two 10-min apart blood samples were drawn before a 75-g oral glucose tolerance test (OGTT). Then, blood samples were collected at 15, 30, 60, 90, 120 and 180 min for glucose, lactate (every 30 min), FFA, insulin and C-peptide concentrations. In addition, chemokine (fractalkine, MCP1 [monocyte chemoattractant protein 1], IL6 [interleukin 6], IL8 [interleukin 8] and RANTES [regulated on activation, normal T cell expressed and secreted]) blood concentrations were measured at fasting and 60 min after the OGTT. Gas exchange was also measured during the last 20 min of each hour after the OGTT. At 180 min, a second 75-g OGTT was performed following the same procedures, except that chemokines were not measured.

### Resting metabolic rate and carbohydrate oxidation

On each testing day, the flow sensor was calibrated with a 3-L syringe; and the analyzers were calibrated with standardized gases (16% O_2_/4% CO_2_ and 26% O_2_) before each gas exchange assessment. $\dot{V}{\text{O}}_{\text{2}}$ and $\dot{V}{\text{CO}}_{\text{2}}$ were calculated at 30 sec intervals. After gas exchange assessment, a post-calorimetric adjustment of $\dot{V}{\text{O}}_{\text{2}}$ and $\dot{V}{\text{CO}}_{\text{2}}$ was performed using high-precision mass-flow regulators (series 358; 0–2 L/min; Analyt-MTC [Müllheim, Germany]) [19]. Metabolic rate was calculated after standardization for temperature, pressure and moisture. Non-protein respiratory quotient (npRQ) was calculated as the $\dot{V}{\text{CO}}_{\text{2}}$ to $\dot{V}{\text{O}}_{\text{2}}$ ratio after considering an estimated urinary nitrogen excretion rate of 0.5 g/h. Carbohydrate oxidation was determined from previously published equations [20].

### Serum/plasma biochemical analysis

Plasma glucose and lactate were measured by the glucose and lactate oxidase methods, respectively. Serum FFA concentration by an enzymatic colorimetric method (NEFA-HR. Wako Chemicals. Richmond, VA), serum insulin by direct chemiluminescent technology (Advia Centaur. Bayer Corp. Newbury, UK) and serum C-peptide by chemiluminescent technology (Immulite. Siemens. Erlangen, Germany). Plasma IL6, IL8, MCP1 and RANTES were measured by Magnetic Luminex Screening Assay (R&D Systems. Minneapolis, USA); and plasma fractalkine by ELISA (Quantikine; R&D Systems. Minneapolis, USA). Blood responses were calculated over each 3-h period by the incremental (for glucose, lactate, insulin, C-peptide, resting metabolic rate, npRQ and carbohydrate oxidation) and total (for FFA) area under the curve (AUC) using the trapezoidal method.

### Insulin sensitivity

Insulin sensitivity was estimated from circulating glucose and insulin concentration at fasting and after oral glucose load through the 3-hour Oral Glucose Insulin Sensitivity index (OGIS) validated against the euglycemic-hyperinsulinemic clamp [20]. The OGIS index can be calculated online (http://webmet.pd.cnr.it/ogis/ogis.php).

### Insulin secretion rate from serum C-peptide concentration analysis

Insulin secretory rate during the two 3-h OGTT periods was calculated by deconvolution of the plasma C-peptide concentration. β cell function parameters were obtained using the model by Mari et al. [21,22]. This model expresses glucose-stimulated insulin secretion (in pmol·min^-1^·m^-2^) as the sum of two components. The first component represents the dependence of insulin secretion on the absolute glucose concentration at any time point during the OGTT and is characterized by a dose-response function (slope = β cell glucose sensitivity). The dose-response is modulated by a potentiation factor that encompasses several glucose-dependent and glucose-independent potentiating mechanisms (e.g., gastrointestinal hormones). In normal individuals, the potentiation factor typically increases from baseline to the end of a 2-h OGTT, which is quantified by the ratio of the mean values at 100–120, 160–180, 280–300 and 340–360 min relative to baseline mean values at 0–20 min post initial OGTT (potentiation factor ratio [PFR]). The second component (rate sensitivity) represents the dependence of insulin secretion on the rate of change of plasma glucose concentration and is proportional to the first derivative of plasma glucose concentration against time. Rate sensitivity accounts for the observation that rapid changes in glucose concentration enhance insulin secretion early in the OGTT.

### Glucose-stimulated insulin secretion in isolated mice pancreatic islets incubated with RANTES and IL8

Adult 8 weeks-old mice were anesthetized with a mix of ketamine:xylazine (0.18 mg:0.012 mg per gram of mouse) by intraperitoneal injection. Pancreas was perfused with collagenase (0.21 mg/mL of Liberase TL [Roche]) through the common bile duct prior to euthanasia (by incision of the chest cavity to produce a bilateral pneumothorax). After verification of death, pancreas was removed out of the animal. Islets were isolated after pancreas digestion (37°C for 14 min), followed by Histopaque^®^ 1077 (Sigma) density gradient separation and handpicked purification. Islets were incubated for 24 h in RPMI 1640 medium containing 11.2 mmol/L glucose, 10% FBS, 110 μg/mL sodium pyruvate, 110 U/mL penicillin and 110 μg/mL streptomycin. Five islets/well were incubated for 24 h with/without 200 ng/mL of human recombinant IL8 or 200 ng/mL of mouse recombinant RANTES/CCL5 (both from R&D System). After incubation, islets were washed for one hour with Krebs-Ringer-Hepes buffer (KRH in mmol/L: 137 NaCl, 4.8 KCl, 1.2 KH_2_PO_4_, 1.2 MgSO_4_, 2.5 CaCl_2_, 5 NaHCO_3_, 16 HEPES and 0.1% BSA) at 2.8 mmol/L glucose and supernatant was eliminated. Then, islets were incubated for one hour with KRH 2.8 mmol/L glucose followed by one hour at 16.7 mmol/L glucose. Incubations were performed at 37°C and 5% CO_2_. Supernatants were collected, while islets were lysed in HCl-ethanol. Supernatants and lysates were stored at -20°C until insulin determination (ELISA, Merck-Millipore). Insulin secretion is expressed as a percentage of total insulin content.

### Statistical analysis

Data are presented as mean ± SD or SE. Analyses were performed using SAS version 9.2 (SAS Institute, Cary, NC, USA). Repeated-measures ANOVA and post-hoc Tukey analyses were conducted to assess differences with time. Associations between variables were analyzed by Spearman rank correlations. Power analysis showed that 53 subjects will be sufficient to detect a correlation of 0.38 or higher. Stepwise multiple regression analysis was also conducted to identify the factors best predicting insulin secretion rate over the whole 6-h and first 3-h periods. Variables with a variance inflation factor equal or greater than 10 were excluded from the analysis. Influence of sex, age, body mass index and insulin sensitivity on insulin secretion rate was initially tested. Further analysis included carbohydrate oxidation, circulating lactate, FFA, and chemokines/cytokines concentration. The significance level was set at p<0.05.

## Results

### Blood metabolite and hormonal response

**Fig 1:**
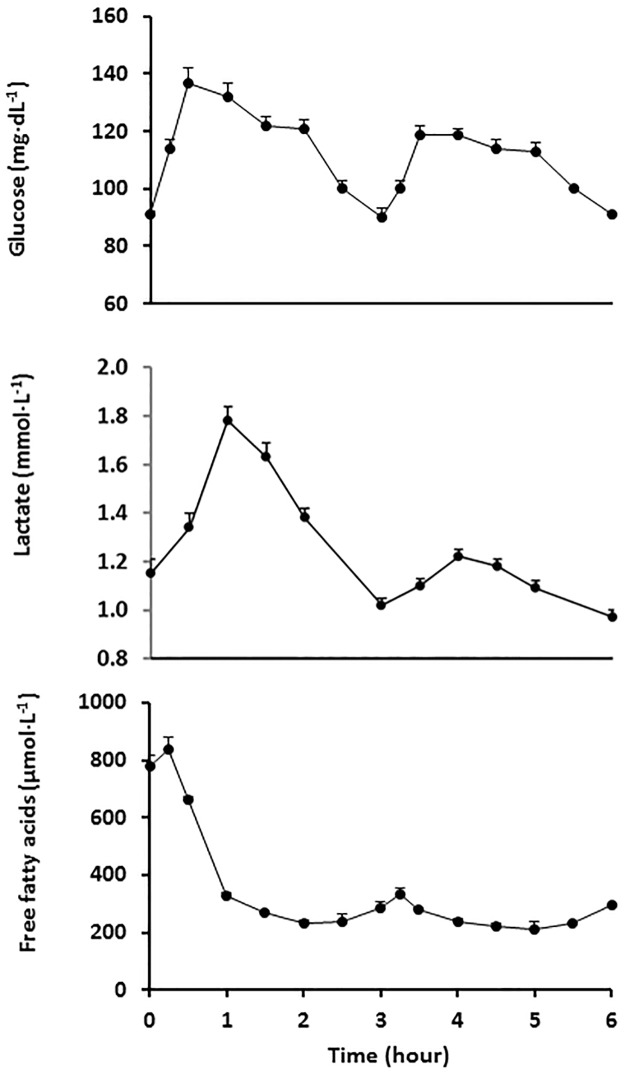
Circulating glucose, lactate and free-fatty acid concentrations before and after two consecutive 75-g oral glucose loads. Glucose loads were given at time 0 and 3 h. Mean±SE.

Upon oral glucose ingestion, circulating glucose concentration increased up to 137±24 mg·dL^-1^ at 30 min and returned to fasting values after 150 min (100±22 mg·dL^-1^; p = 0.25). After the second glucose load, it reached a peak between 30 and 60 min (119±22 and 119±24 mg·dL^-1^, respectively) although at a lower extent when compared with values after the first glucose loading (p<0.001). The integrated response assessed as the incremental area under the curve was 86.5±52.7 and 62.3±30.1 mg·h·dL^-1^ over the first and second glucose loading, respectively (p<0.001; Fig 1A). Circulating lactate concentration followed a profile similar to glucose, with a postprandial integrated response after the second versus first glucose loading much more attenuated (28.0±32.7 and 58.8±41.3 mmol·min·L^-1^, respectively; p<0.00001) (Fig 1B). Circulating FFA concentration was strongly suppressed after oral glucose ingestion, and it remained low over the entire period, with no further suppression after consecutive glucose loading (Fig 1C).

**Fig 2:**
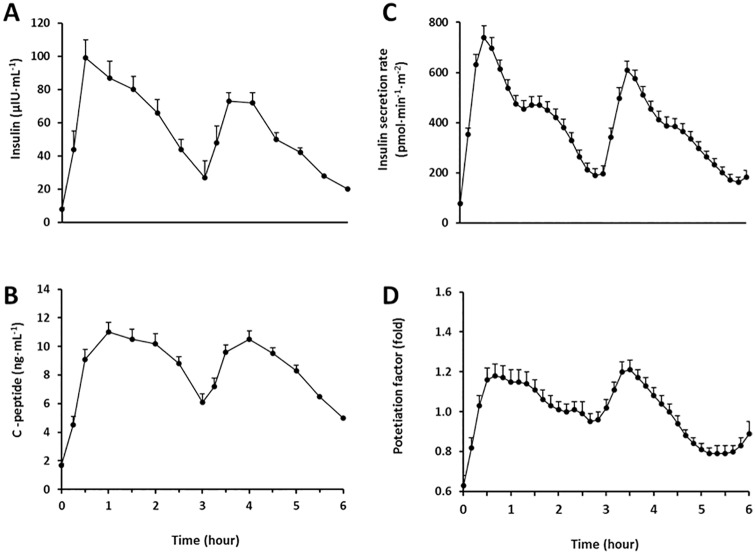
Serum insulin and C-peptide concentrations before and after two consecutive 75-g oral glucose loads. Glucose loads were given at time 0 and 3 h. Mean±SE.

After glucose ingestion, circulating insulin concentration increased up to 18±14 fold and remained elevated ~4-fold at the end of the first period when compared with fasting value. Upon the second glucose loading, it increased at a lower extent relative to fasting value (14±7 fold; p = 0.001). Consistently, the area under the curve was lower after the second vs. first glucose loading (119±71 vs. 169±91 μIU·h·mL^-1^, respectively; p<0.0001; Fig 2A). In turn, circulating C-peptide concentration increased up to 8±2 fold after the first glucose load, and it achieved a similar fold increase after the second glucose load relative to fasting value (7±2 fold; p = 0.11). The integrated response was similar between the first and second period (21.3±8.1 and 22.3±15.1 ng·h·mL^-1^, respectively; p = 0.53; Fig 2B).

### Insulin sensitivity, insulin secretion and β cell function

Insulin secretion rate was decreased after the second vs. first glucose load (p<0.0001; Fig 2C). The mean increase in potentiation factor during the second and first 3-h periods were similar (1.04±0.15 and 0.96±0.15, respectively; p = 0.08; Fig 2D). Total insulin secretion over 6 hours was inversely related with insulin sensitivity estimated by OGIS (r = -0.55; p<0.0001), as expected. The β cell function parameters are shown in Table 2. None of these parameters related with OGIS, except for a borderline inverse association with potentiation factor ratio at the end of the first 3-h glucose load period (r = -0.26; p = 0.07).

**Table 2:** Insulin secretion and β-cell function parameters.

	Mean ± SD	Range
Basal insulin secretion (pmol·min^−1^·m^−2^)	76.6 ± 28.4	34–172
OGTT Insulin secretion 0–180 min (nmol·m^-2^)	78.5 ± 29.2	31.5–145.0
OGTT Insulin secretion 180–360 min (nmol·m^-2^)	63.8 ± 25.2	28.9–145.0
OGTT Insulin secretion 0–360 min (nmol·m^-2^)	142 ± 52	63–277
Glucose sensitivity (pmol·min^−1^·m^−2^·mM^−1^)	156 ± 63	59–311
Rate sensitivity (pmol·m^−2^·mM^−1^)	1327 ± 846	0–2974
Potentiation factor ratio 120	1.43 ± 0.73	0.09–4.36
Potentiation factor ratio 180	1.35 ± 0.73	0.47–4.65
Potentiation factor ratio 300	0.80 ± 0.26	0.28–1.42
Potentiation factor ratio 360	0.80 ± 0.36	0.28–2.10

Potentiation factor ratio calculated as the ratio between the mean potentiation factor at 100–120, 160–180, 280–300 and 340–360 min and the mean baseline potentiation factor (0–20 min) after the initial OGTT.

### Blood chemokine response

**Fig 3:**
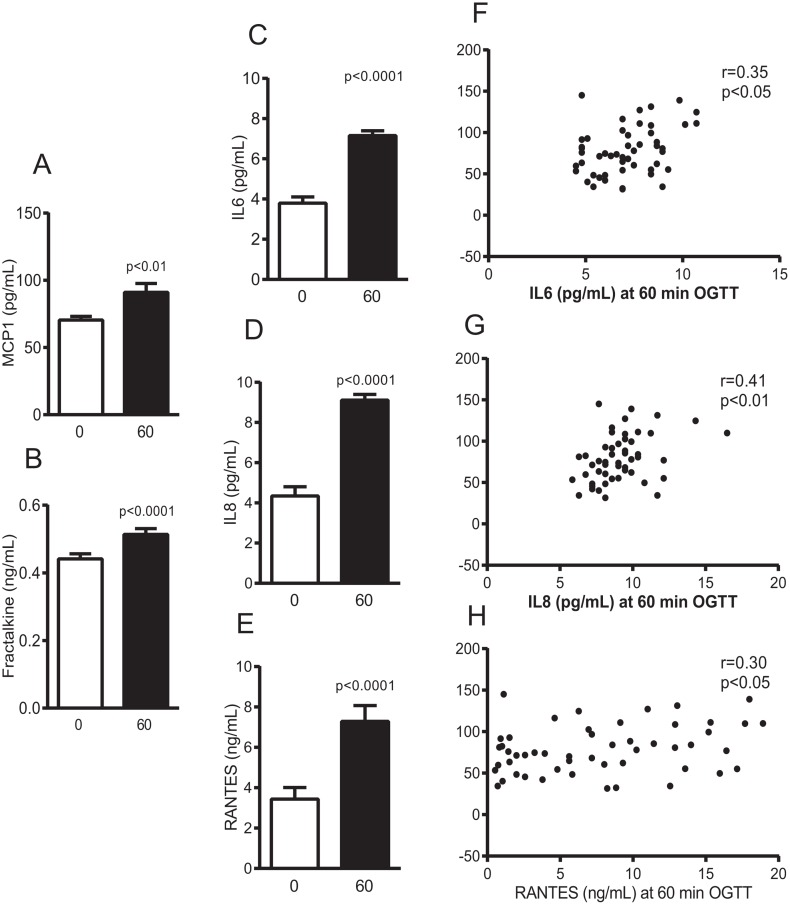
Plasma chemokine concentrations before and at 60 min after a 75-g oral glucose load (A-E) and Spearman correlations of IL6 (F), IL8 (G) and RANTES (H) with insulin secretion rate. Mean±SE.

Circulating chemokines increased upon oral glucose stimulation (Fig 3A–3E), with an average increase of 1.2±0.3 fold for fractalkine and 5.8±7.0 fold for RANTES. The chemokine levels, evaluated at 60 min after the oral glucose load or as the fold change relative to fasting, were not related with carbohydrate oxidation over the first glucose load period.

### Metabolic rate, fuel partitioning and carbohydrate oxidative response

**Fig 4:**
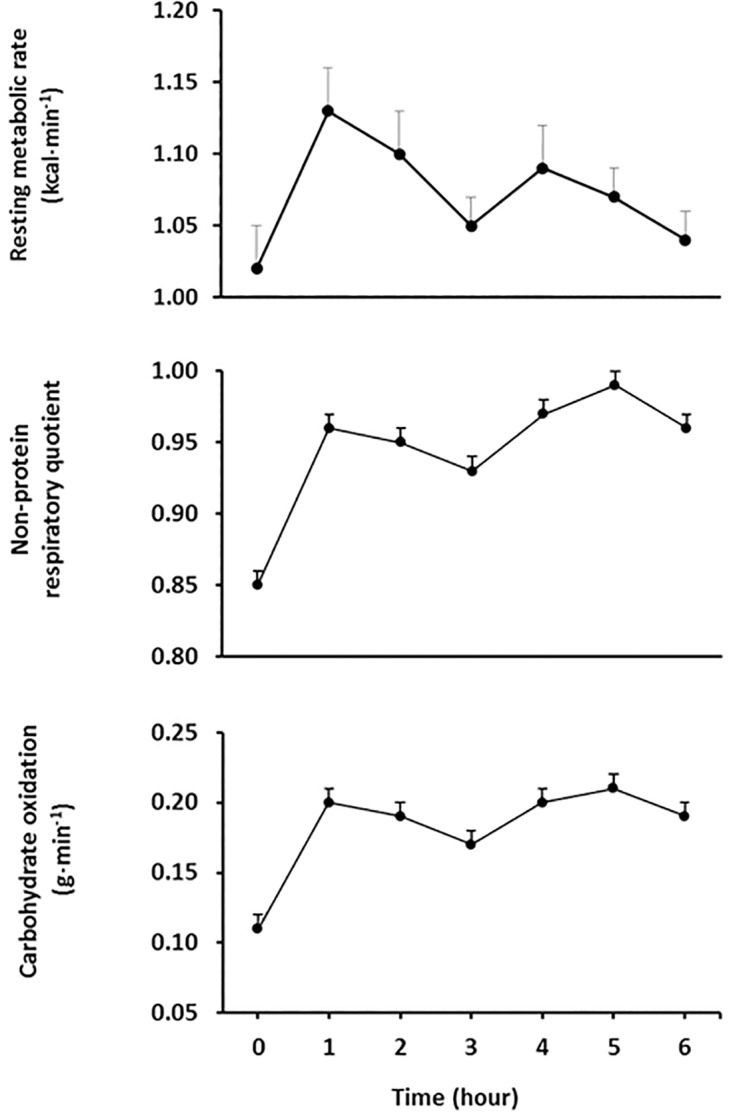
Resting metabolic rate, non-protein respiratory quotient and carbohydrate oxidation before and after two consecutive 75-g oral glucose loads. Glucose loads were given at time 0 and 3 h. Mean±SE.

Under fasting conditions, metabolic rate was 1.02±0.17 kcal·min^-1^ and increased after oral glucose loading up to 1.13±0.18 kcal·min^-1^ at 60 min (p<0.0001). At the end of the 3-h period, metabolic rate was 1.05±0.15 kcal·min^-1^, which showed a borderline difference with fasting metabolic rate (p = 0.05). After 60 min of the second glucose load, metabolic rate increased at a lower extent (1.09±0.19 kcal·min^-1^), being this value lower than the magnitude observed after 60 min of the first glucose load (p = 0.02; Fig 4A). Consistently, the second vs. first integrated response was lower (11.0±8.5 and 12.7±8.3 kcal·min·min^-1^, respectively; p = 0.02). Postprandial npRQ showed a pattern consistent with increased carbohydrate oxidation as anticipated when glucose is the only exogenous carbon source. In addition, npRQ was higher after the second vs. first glucose load as indicated by the integrated response (22.3±10.9 and 15.8±9.5, respectively; p<0.0001; Fig 4B). In line with npRQ, carbohydrate oxidation increased after glucose loading and remained high for the entire period (p<0.0001; Fig 4C). After the second vs. first glucose loading, maximal carbohydrate oxidation showed a borderline significant increase (0.238±0.010 and 0.221±0.009 g·min^-1^, respectively; p = 0.052). The integrated carbohydrate oxidative response after the first and second glucose load were 12.7±6.7 and 16.7±8.0 g·min·min^-1^, respectively (p<0.0001).

### Relationship between insulin sensitivity and secretion: role of carbohydrate oxidation

Insulin sensitivity was not associated with carbohydrate oxidation during the whole test (r = 0.07; p = 0.65) nor after the first glucose loading (r = 0.07; p = 0.65), while over the second postprandial period reached borderline significance (r = -0.27; p = 0.06). Insulin secretion over 6 h did not relate with 6-h carbohydrate oxidation (r = -0.11; p = 0.45) or its respective 3-h periods (p = 0.27–0.45). Similarly, 6-h carbohydrate oxidation did not associate with β cell function indexes such as glucose sensitivity (r = 0.02; p = 0.88), rate sensitivity (r = -0.07; p = 0.61) or any of the potentiation factor ratios (p = 0.42–0.84).

### Role of circulating chemokines, lactate and FFA concentrations on insulin secretion

Insulin sensitivity was not associated with circulating chemokine concentrations (Table 3). In turn, insulin secretion rate during the first period related directly with plasma IL8, IL6 and RANTES determined after 60 min of glucose loading (Fig 3F–3H). Such associations remained similar or even higher when adjusted for insulin sensitivity. In addition, the association of circulating chemokines with β cell function parameters was also examined. Only plasma IL8 concentration at 60 min OGTT related with β cell glucose sensitivity (r = 0.29; p = 0.04) (Table 3).

**Table 3:** Spearman correlation matrix of circulating chemokines concentrations with insulin sensitivity and β-cell function after the first 75-g oral glucose load.

	Fractalkine			MCP1			IL6			IL8			RANTES		
	Fasting	60	δ	Fasting	60	δ	Fasting	60	δ	Fasting	60	δ	Fasting	60	δ
**Insulin sensitivity**															
OGIS	-0.06	-0.01	0.03	0.05	0.00	-0.02	0.01	-0.14	-0.02	0.11	-0.08	-0.13	0.06	-0.12	-0.07
**Insulin secretion rate**															
Basal	-0.17	-0.18	-0.02	-0.10	0.11	0.13	-0.03	0.15	0.12	-0.06	0.27	0.22	-0.05	0.09	0.06
OGTT	-0.02	-0.02	-0.07	-0.16	0.08	0.18	0.02	**0.35**	0.18	0.11	**0.41**	0.15	-0.09	**0.30**	0.23
**β-cell function indexes**															
GS	0.03	0.11	0.05	-0.15	0.17	0.17	0.06	0.18	0.03	0.13	**0.29**	0.03	0.11	0.21	0.11
RS	0.02	-0.06	-0.08	-0.16	-0.18	-0.04	-0.11	0.02	0.09	-0.13	0.01	0.09	-0.21	-0.01	0.10
PFR_120_	0.03	0.14	0.15	-0.18	0.08	0.15	-0.07	0.01	0.10	-0.18	0.00	0.21	-0.12	-0.01	-0.03
PFR_180_	0.18	0.03	-0.05	0.00	0.18	0.11	-0.18	0.06	0.21	-0.21	-0.01	0.23	-0.14	-0.02	0.08

60 = 60 min after oral glucose load (OGTT); δ = Chemokine concentration at 60 min–fasting values; GS, β-cell glucose sensitivity; RS, rate sensitivity; PFR calculated as the ratio between the mean potentiation factor at 100–120 (PFR_120_) and 160–180 (PFR_180_) and the mean baseline potentiation factor (0–20 min) after the first OGTT. Values in bold p<0.05

With regard to lactate and FFA, no association was detected between the 6-h integrated response for lactate (r = 0.14; p = 0.32) and FFA (r = -0.21; p = 0.13) with insulin sensitivity (by OGIS after the first glucose load). However, an inverse correlation between the integrated response for FFA after the first glucose period and insulin sensitivity was detected (r = -0.40; p<0.01). While, 6-h carbohydrate oxidation related directly with lactate (r = 0.40; p<0.01) but not FFA (r = -0.05; p = 0.72) integrated responses. In turn, the integrated 6-h FFA (r = -0.01; p = 0.94) and lactate (r = -0.02; p = 0.91) responses did not relate with 6-h insulin secretion rate. Further analysis found that 6-h lactate response associated with potentiation factor ratio at the end of the second glucose load (r = -0.35; p = 0.01), while a borderline significant correlation was observed for rate sensitivity (r = 0.25; p = 0.07) (S1 Table).

### Multiple regression analysis of insulin secretion rate

Initial analysis including insulin sensitivity (OGIS over the first period), body mass index, age and sex showed that only insulin sensitivity explained part of the variance in 6-h insulin secretion rate (R^2^ = 0.25; p<0.001). Further analysis considered the integrated response over the 6-h period (i.e. AUC) for carbohydrate oxidation, lactate and FFA and chemokines/cytokines (at 60 min OGTT). This analysis showed that besides insulin sensitivity, IL8 was also an independent factor associated with insulin secretion rate (adjusted R^2^ = 0.31; p<0.0001; Table 4). Similar analysis conducted for the first 3-h period showed that insulin sensitivity (OGIS over the first period), IL8 (at 60 min OGTT) and also age explained about 40% of the variance in insulin secretion rate (adjusted R^2^ = 0.39; p<0.0001; Table 4).

**Table 4:** Multiple regression model of glucose-stimulated insulin secretion.

	Partial R^2^	Total R^2^	β	p
**6-h period**				
**Intercept**			227±48	<0.0001
**Insulin sensitivity**	0.25	0.25	-0.38±0.09	0.0001
**IL8 at 60 min OGTT**	0.09	0.34	7.9±3.1	0.01
**First 3-h period**				
**Intercept**			75±29	0.01
**Insulin sensitivity**	0.21	0.21	-0.19±0.05	<0.001
**IL8 at 60 min OGTT**	0.13	0.34	5.3±1.6	<0.01
**Age**	0.08	0.42	0.97±0.38	0.01

Insulin sensitivity determined by OGIS after the first OGTT.

### Effect of RANTES and IL8 on glucose-stimulated insulin secretion from isolated mice islets

**Fig 5:**
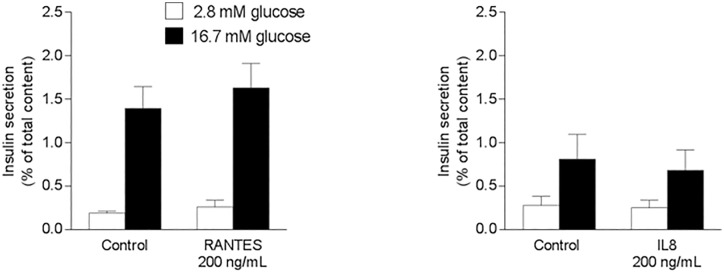
Glucose-stimulated insulin secretion in isolated mice islets incubated with recombinant RANTES or IL8. Glucose-stimulated insulin secretion in isolated pancreatic islets after incubation for 24 h with/without 200 ng/mL RANTES/CCL5 (A) or IL8 (B). n = 6–8 experiments. Mean±SE.

Glucose-stimulated insulin secretion was similar between the control and RANTES-treated (1.4±0.2 vs. 1.6±0.3%, respectively; p = 0.64) or IL8-treated (0.8±0.3 vs. 0.7±0.2%, respectively; p = 0.88) conditions (Fig 5). No differences in basal insulin secretion were detected.

## Discussion

This cross-sectional study did not find an association between whole-body carbohydrate oxidation and insulin secretion or β cell function. We postulated that such putative interaction between carbohydrate oxidation and insulin secretion might be mediated through skeletal muscle-released circulating factors. Among these factors, carbohydrate oxidation only related with plasma lactate concentration; however, this metabolite did not associate with insulin secretion rate. In turn, three out of 5 chemokines assessed related directly with insulin secretion rate, but none of them associated with carbohydrate oxidation or insulin sensitivity.

Our hypothesis that carbohydrate oxidative disposal, particularly in skeletal muscle, may modulate insulin secretion is mainly grounded on three observations. First, mice genetic models featured by altered skeletal muscle glucose metabolism have abnormal *in vivo* insulin secretion when compared with wild-type animals [8,9]. Second, findings in human that highlights a role of insulin stimulating its own *in vivo* insulin secretion may actually be a consequence of the expected change in insulin-mediated skeletal muscle glucose metabolism [10,11]. Such explanation is reinforced after considering that insulin has often been reported to inhibit *in vitro* insulin secretion [13–16]. Finally, we recently found that 24-h carbohydrate oxidation and 24-h insulin secretion are inversely related, which was independent of insulin sensitivity [17]. This later finding may support a putative interaction between pancreas and distant organs, considering that most of the whole-body carbohydrate oxidation proceeds in peripheral tissues, including skeletal muscle. Taken together, skeletal muscle might release factors into circulation that are sensitive to fluctuations in glucose metabolism, which will then modify insulin secretion. This idea offers a mechanism to explain the well-reported inverse association between insulin sensitivity and secretion [1]. However, our study did not support a role of carbohydrate oxidation driving the release of circulating factors affecting insulin secretion.

It must be acknowledged that whole-body carbohydrate oxidation may not be representative of skeletal muscle oxidative glucose disposal under a non-steady-state (i.e. postprandial) condition, thereby preventing to detect an eventual glucose flux-sensitive muscle-pancreas crosstalk. Assessment of *in vivo* skeletal muscle carbohydrate oxidation and myokine secretion through limb balance technique offers an optimal model to test this hypothesis. Lack of support of this study to our hypothesis may also lie on a greater influence of other peripheral organs, such as liver, on insulin secretion. In this regard, specific liver-secreted factors have action on pancreas, specifically by promoting β cell proliferation [23,24]. Adipose tissue might also have a role on pancreas, for instance, through FFA. Here we observed no association between circulating FFA concentration and insulin secretion. That finding is consistent with an *in vivo* human study that found similar glucose-stimulated insulin secretion after a 4-h insulin pre-infusion at low and high serum FFA levels (with/without Intralipid/heparin) [25].

Besides FFA as potential mediators, we considered specific chemokines known to be secreted from muscle cells as potential candidates [7,26]. IL6 and fractalkine have shown to influence insulin secretion both in animals and humans [8,27–29]. In turn, IL8, RANTES and MCP1 have been detected in human muscle cell-derived conditioned media [7,30], and their receptors are expressed in human β cells [31,32]. The extent at which skeletal muscle contributed to circulating chemokine concentration cannot be ascertained in this study. Other tissues and immune cells may certainly contribute. A previous study estimated through forearm balance that ~10% of systemic IL6 concentration comes from skeletal muscle [33]. Interestingly, all measured chemokines increased their circulating concentration upon oral glucose stimulation. Our finding is consistent for IL8, but inconsistent for IL6 and MCP1 [34,35], while glucose-stimulated circulating RANTES and fractalkine concentrations have not been reported. It is remarkable that the glucose-stimulated increase in plasma IL6 concentration is within the reported elevation after an acute bout of exercise [36].

As aforementioned, no associations between carbohydrate oxidation and plasma chemokine concentration were found. However, IL6, RANTES and IL8 correlated directly with insulin secretion rate, even after adjusting for insulin sensitivity. Although IL6 has been independently associated with insulin secretion [28] as well as having a direct effect on *in vivo* and *in vitro* insulin secretion [8,37,38], in our present study IL6 was not an independent determinant of insulin secretion. In turn, RANTES has shown to stimulate *in vitro* insulin secretion from mouse and human islets [32]. In contrast, we did not confirm RANTES as an independent determinant of *in vivo* insulin secretion or having an action on *in vitro* insulin secretion. Considering IL8 as an independent predictor of insulin secretion and the only chemokine associated with β cell glucose sensitivity prompted the idea that IL8 may be a candidate regulating insulin secretion. The opportunity to assess the direct effect of human recombinant IL8 on insulin secretion from isolated islets counterbalanced the fact that mice have deleted the gene encoding IL8 [39]. At some extent, one can still expect a biological action considering that chemokines often share their receptors. However, our *in vitro* assay did not support a role of IL8 on insulin secretion.

Lactate may also be a potential mediator factor considering that skeletal muscle plays a predominant role on its metabolism [40]. In addition, we [41] and others [42] have found a direct correlation between plasma lactate concentration and impaired insulin sensitivity. Moreover, lactate can stimulate *in vitro* insulin secretion [43,44]. Thus, under the context of our hypothesis, lactate was a good candidate linking insulin sensitivity with insulin secretion. However, in this study we did not confirm the association between circulating lactate concentration and insulin resistance. Furthermore, plasma lactate concentration did not correlate with insulin secretion, although it did show (6-h lactate integrated response) an inverse association with the potentiation factor ratio at the end of the second glucose load. The relevance of this time-specific finding is uncertain.

The extent at which glucose reaches circulation may also play a role determining insulin secretion. Indeed, a previous study using a similar design (two consecutive 3-h apart 75-g OGTTs) found that on average 80% of the ingested glucose appeared in circulation over the first 3-h period [18]. Such absorption rate showed a between-subject variability of 42%. Thus, insulin secretion rate may well be partially influenced by this process.

In conclusion, classically reported inverse association between insulin sensitivity and its secretion was unaccounted by whole-body carbohydrate oxidation. Further assessment of this hypothesis will require determination of skeletal muscle glucose oxidation and its secreted factors. Manipulation of carbohydrate oxidation may also be informative, although difficult to interpret considering that carbohydrate oxidation is tightly involved in ATP synthesis, which can affect other critical physiological outcomes. IL8 seems a potential circulating factor with action on insulin secretion and β cell function. Systemic intravascular infusion or incubation of isolated human pancreatic islets or β cells with IL8 will elucidate the relevance of our correlational finding. Alternative studies can also take advantage of an *in vitro* model intended to modify glucose metabolism in muscle cells. The influence of the conditioned media on insulin secretion can then be assessed. These studies will translate animal findings [8,9] to humans as well as identifying the mechanism mediating the association between insulin sensitivity and its secretion.

## Supporting Information

S1 Table Spearman correlation matrix of circulating 6-h integrated FFA and lactate responses with β-cell function. PFR, potentiation factor ratio calculated as the ratio between the mean potentiation factor at 100–120, 160–180, 280–300 and 340–360 min and the mean baseline potentiation factor (0–20 min) after the initial OGTT. *p = 0.07; values in bold p<0.05.
(DOCX)

## Abbreviations

AUC: area under the curve
FFA: free-fatty acids
FFM: fat-free mass
IL6: interleukin 6
IL8: interleukin 8
MCP1: monocyte chemoattractant protein 1
npRQ: non-protein respiratory quotient
OGIS: oral glucose insulin sensitivity
OGTT: oral glucose tolerance test
PFR: potentiation factor ratio
RANTES: regulated on activation, normal T cell expressed and secreted
