# Supplementary material for: Assessment of the Role of Metabolic Determinants on the Relationship between Insulin Sensitivity and Secretion
Source: PLoS One. 2016 Dec 21;11(12):e0168352. doi: 10.1371/journal.pone.0168352 (PMC5176173; doi:10.1371/journal.pone.0168352)
Supplement: S1 Table — PFR, potentiation factor ratio calculated as the ratio between the mean potentiation factor at 100–120, 160–180, 280–300 and 340–360 min and the mean baseline potentiation factor (0–20 min) after the initial OGTT. *p = 0.07; values in bold p<0.05. (DOCX) [file pone.0168352.s001.docx]

**S1 Table. Spearman correlation matrix of circulating 6-h integrated FFA and lactate responses with β-cell function.**

PFR, potentiation factor ratio calculated as the ratio between the mean potentiation factor at 100-120, 160-180, 280-300 and 340-360 min and the mean baseline potentiation factor (0-20 min) after the initial OGTT.

*p=0.07; values in bold p<0.05.

|  | **6-h FFA** | **6-h lactate** |
| --- | --- | --- |
| **Glucose sensitivity** | -0.05 | -0.02 |
| **Rate sensitivity** | 0.00 | 0.25* |
| **PFR 120** | -0.01 | 0.16 |
| **PFR 180** | 0.03 | 0.05 |
| **PFR 300** | -0.14 | 0.03 |
| **PFR 360** | 0.21 | **-0.35** |
